# Aerobic exercise protects MI heart through miR-133a-3p downregulation of connective tissue growth factor

**DOI:** 10.1371/journal.pone.0296430

**Published:** 2024-01-25

**Authors:** Niu Liu, Zhiping Zhen, Xin Xiong, Yaqi Xue

**Affiliations:** 1 College of P.E, Beijing Normal University, Beijing, China; 2 School of Physical Education, Weinan Normal University, Weinan, Shaanxi, China; Jordan University of Science and Technology Faculty of Medicine, JORDAN

## Abstract

**Objective:**

To investigate the effect of aerobic exercise intervention to inhibit cardiomyocyte apoptosis and thus improve cardiac function in myocardial infarction (MI) mice by regulating CTGF expression through miR-133a-3p.

**Methods:**

Male C57/BL6 mice, 7–8 weeks old, were randomly divided into sham-operated group (S group), sham-operated +aerobic exercise group (SE group), myocardial infarction group (MI group) and MI + aerobic exercise group (ME group). The mice were anesthetized the day after training and cardiac function was assessed by cardiac echocardiography. Myocardial collagen volume fraction (CVF%) was analyzed by Masson staining. Myocardial CTGF, Bax and Bcl-2 were detected by Western blotting, and myocardial miR-133a-3p was measured by RT-qPCR.

**Results:**

Compared with the S group, miR-133a-3p, Bcl-2 and EF were significantly decreased and CTGF, Bax, Bax/ Bcl-2, Caspase 3, Cleaved Caspase-3, LVIDd, LVIDs and CVF were significantly increased in the MI group. Compared with the MI group, miR-133a-3p, Bcl-2 and EF were significantly increased, cardiac function was significantly improved, and CTGF, Bax, Bax/ Bcl-2, Caspase 3, Cleaved Caspase-3, LVIDd, LVIDs and CVF were significantly decreased in ME group. The miR-133a-3p was significantly lower and CTGF was significantly higher in the H2O2 intervention group compared with the control group of H9C2 rat cardiomyocytes. miR-133a-3p was significantly higher and CTGF was significantly lower in the AICAR intervention group compared to the H_2_O_2_ intervention group. Compared with the control group of H9C2 rat cardiomyocytes, CTGF, Bax and Bax/Bcl-2 were significantly increased and Bcl-2 was significantly decreased in the miR-133a-3p inhibitor intervention group; CTGF, Bax and Bax/Bcl-2 were significantly decreased and Bcl-2 was significantly upregulated in the miR-133a-3p mimics intervention group.

**Conclusion:**

Aerobic exercise down-regulated CTGF expression in MI mouse myocardium through miR-133a-3p, thereby inhibiting cardiomyocyte apoptosis and improving cardiac function.

## Introduction

Myocardial ischemia and hypoxia after myocardial infarction (MI) lead to oxidative stress, apoptosis, ventricular malignant remodeling, inflammatory response and deterioration of cardiac function, eventually developing into heart failure [1–3]. Physical exercise is one of the most important tools for cardiovascular disease prevention and rehabilitation [4], and appropriate exercise can inhibit myocardial apoptosis [5, 6], improve myocardial pathological remodeling [7], promote revascularization [8], reduce myocardial fibrosis [9], and improve left ventricular systolic dysfunction [10]. Aerobic exercise can maximize the cardioprotective effects of exercise [10], but its targets and mechanisms of action have not been elucidated.

MicroRNAs (miRs) are small (18–25 nucleotides in length), single-stranded non-coding RNAs that act as post-transcriptional regulators of gene expression through incomplete base pairing with complementary sequences in the 3′-untranslated region. MiRNAs play a more important regulatory role in processes such as cardiovascular development and disease development in mammals. Currently, the bulk of national and international studies have shown that a variety of miRNAs play important roles in myocardial remodeling. Muscle and/or heart-specific miRNAs, miR-1, miR-133a, miR-133b, and miR-208 are involved in cardiac development and cardiovascular diseases, including MI [11]. It was found that miR-133a is protective against myocardial fibrosis and has been shown to regulate cardiac development [12–14] and is dysregulated in hypertrophied and failing hearts [15–17]. Studies have shown that microRNAs can regulate the control of myocardial remodeling from multiple transmission pathways such as connective tissue growth factor (CTGF), angiotensin system and inflammatory factors [18, 19].

Connective tissue growth factor (CTGF), also known as CCN2, belongs to the CCN (an acronym for Cyr61, CTGF, and Nov) family of stromal cell proteins and is a 38 kDa cellular matrix-inducible stromal cell protein with a motif consisting of five exons that is highly conserved in vertebrates [20]. During AF-induced atrial fibrosis, pro-fibrotic factors including angiotensin II (Ang II) and transforming growth factor-β1 (TGF-β1) regulate the expression of connective tissue growth factors. CTGF exerts an inducible effect on atrial fibrosis [21]. In various cardiac diseases, CTGF acts as a cardiac autocrine factor involved in regulating various important biological functions, including angiogenesis, apoptosis and fibrosis, as well as other pathological processes in various cardiac diseases [22–26]. CTGF has been reported to be a target of miR-133a and negatively correlated with miR-133a expression [27–29]. There is a lack of literature on whether exercise, a cost-effective means of preventing and treating infarcted hearts, can affect myocardial CTGF factor expression in infarcted mice and what the specific mechanisms are. Therefore, in this paper, we investigated the protective effect of aerobic exercise on infarcted heart function by regulating miR-133a and further influencing CTGF expression, which provides a new experimental and theoretical basis for an in-depth study of the pathological development process of the infarcted heart and offers possible targets and ideas for exploring screening tools and methods for myocardial infarction prevention and treatment.

## Materials and methods

### Animals

Male C57BL/6J mice, 3~4 weeks old, weighing 20 g±1.5 g, were purchased from the Laboratory Animal Center of Xi’an Jiaotong University [Xi’an, China; No: SCXK (Shann) 2017–003]. All animal experiments were approved by the Animal Care and Use Committee of Beijing Normal University and complied with the National Institutes of Health Guide for the Care and Use of Laboratory Animals. Mice were housed in groups in isolation cages on individually ventilated racks lined with poplar shavings, and the cages were sanitized every two weeks. Reverse osmosis water, acidified water and standard autoclaved rodent chow were available for mice to drink ad libitum. Environmental conditions were maintained at constant temperature (22 ± 2°C), standard light/dark cycle with light hours from 8 a.m. to 8 p.m., relative humidity (55 ± 5%). All experimental animals were humanely euthanized using isoflurane anesthesia followed by neck breaking in accordance with American Veterinary Medical Association guidelines.

**Fig 1:**
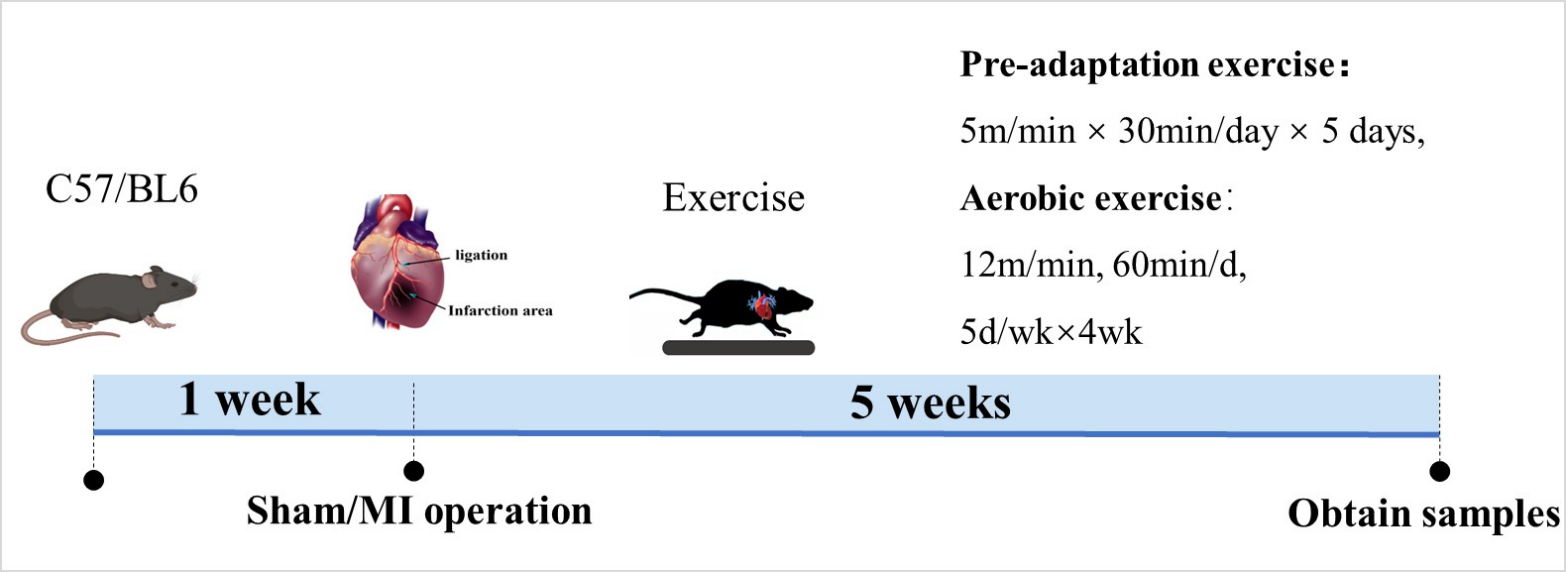
Experimental overview diagram for this article. One week after myocardial infarction surgery, all mice in the exercise group underwent aerobic exercise training on a small animal treadmill for 5 weeks. After exercise, all experimental mice were anesthetized using isoflurane and then subjected to neck-breaking execution in order to collect cardiac tissue and serum samples for subsequent experiments.

As shown in Fig 1, myocardial infarction model was established by ligation of the left anterior descending branch (LAD) of the coronary artery after 1 week of acclimatization. Mice were anesthetized with isoflurane (2% inhalation) on the operating table; the chest cavity was opened, and heart was extruded from the chest. After ligating the left coronary artery below the left auricle with a No. 5/0 silk thread, then, the heart was returned to its original position and the wound was sutured. Successful myocardial infarction was indicated by ST-segment elevation and T-wave inversion on the electrocardiogram. On the seventh postoperative day, echocardiography was performed to assess the extent of MI. Mice in which the left anterior descending branch of the coronary artery was not ligated served as a control group (sham-operated group, S). In this study, animals were randomized into four groups: sham-operated exercise group (SE), sham-operated infarction group (S), infarction group (MI), and infarction aerobic exercise group (ME), with ten mice in each group.

### Ethics approval and consent to participate

The experimental protocols involved in this paper were referred to the Guide to Animal Experiments. All animal experimental designs involved in this study were conducted in accordance with the principles of substitution, reduction and optimization. Moreover, the animal experiment implementation plan must comply with the requirements of Laboratory animal—Guideline for ethical review of animal welfare in China (GB/T 35892–2018), and were approved by the ethical committee of Shaanxi Normal University (Approval number: 20220619001‬; Tile: Aerobic exercise protects MI heart by inducing miR-133a-3p to downregulate CTGF; Date: 19 June 2022).

### Exercise protocol

Experimental exercise protocol was determined based on preliminary experiments (Fig 1): SE and ME underwent pre-adaptive exercise (5 m/min, 30 min/day x 5 days/week) 1 week after infarction surgery. Aerobic exercise intensity was 12 m/min x 60 min, 60 min/day x 5 days/week x 4 weeks. Exercise was performed daily from 19:00 to 21:00 h. Groups S and MI did not exercise and were kept quietly for 5 weeks.

### Echocardiographic measurements

After 24 hours of aerobic exercise, echocardiography was performed using an echocardiograph (VINNO 6 VET, VINNO, China). Mice were anesthetized with isoflurane and placed on the operating table. B-mode echocardiography was first used to find the long axis of the left ventricle in mice, and then transferred to M-mode for data acquisition. Cardiac function was assessed by analyzing left ventricular internal diastolic diameters (LVIDd), systolic internal diameters (LVIDs), and ejection fraction (EF). After data collection, the mice were necropsied, immediately, and the hearts were rapidly collected on ice and stored in pre-cooled formaldehyde or liquid nitrogen for subsequent experiments.

### Cell culture and treatment

H9C2 cardiomyocytes were purchased from the cell bank of Basic Medical Cell Center, Institute of Basic Medical Sciences, Chinese Academy of Medical Sciences. The cells were cultured in high sugar DMEM complete medium with 10% fetal bovine serum (FBS) and 1% penicillin-streptomycin double antibody at 37°C in a constant temperature incubator with 5% CO_2_. The medium was changed every two days, and the cell density was about 70%-80% for passaging and experimental intervention. H9C2 cells were treated with H_2_O_2_ at a concentration of 400 μmol/L for 4 hours to construct a cell infarction model. AICAR, an AMPK agonist at an intervention concentration of 1 mmol/L, was used to simulate the exercise environment [30].

### Cell transfection

H9C2 cardiomyocytes were inoculated into 6-well plates at a density of approximately 3 x 10^5^ according to the experimental requirements. When the cells are 80–90% grown, transfection is performed according to the instructions of the Gene Pharma oligo mRNA transfection reagent. Prior to transfection, cells are replaced to ensure that the medium is antibiotic-free.

miR-133a-3p mimics are transfected at a concentration of 100 nmol, miR-133a-3p inhibitor at 50 nmol, and the same concentration for each control. At least 3 replicates were set up for each set of samples. Cells were collected 36 hours after transfection for subsequent experiments.

### Quantitative real-time polymerase chain reaction (*RT-qPCR*)

Total RNA from cardiac tissues was extracted using Trizol reagent (TaKaRa, Japan) according to the protocol. Each of the synthetic cDNA samples with the Takara Prime Script RT reagent kit was loaded into a Light Cycler quantitative reverse transcription polymerase chain reaction (RT–PCR) system (TaKaRa, Japan) to determine the mRNA levels of a selected list of genes. The sequence of the primers used to detect these genes was as follows: miR-133a-3p F: 5′-GCGTTTGGTCCCCTTCAACC-3′; *miR-133a-3p* R: 5′-CAGTGCGTGTCGTGGAGT-3′; *u6* F: 5′-GACATCAAGAAGGTGGTGAAGC-3′; and *u6* R: 5′-TGTCATTGAGAGCAATGCCAGC-3′. The results were quantified as Ct values, and Ct is defined as the threshold cycle of the polymerase chain reaction at which the amplified product is first detected. U6 was employed as an endogenous reference. The mRNA levels were quantified by the 2^-ΔΔct^ method.

### Western blot analysis

After lysing 100 mg of cardiac tissue with lysis buffer (RIPA: PMSF: Phosphatase Inhibitor = 100:1:1), tissue was centrifuged at 12,000 rpm for 15 minutes. The supernatant after centrifugation was pipetted into a new EP tube for protein quantification using the BCA kit. After electrophoretic separation on a 10% SDS-PAGE gel, the proteins were transferred to an NC membrane (300 mA, 1.5 hours). After skim milk containment for 1.5 h, CTGF primary antibody (1:1000; Sigma) was incubated overnight at 4°C, followed by incubation with peroxidase-conjugated secondary antibody for 1.5 h. The primary antibody was incubated for 1.5 h with peroxidase-conjugated secondary antibody and then incubated for 1.5 h with peroxidase-conjugated secondary antibody. Images were processed and analyzed using Image Lab software.

### Masson’s trichrome staining

To assess myocardial collagen deposition, myocardial tissue paraffin sections were stained using Masson trichrome staining in this experiment. Briefly, myocardial tissue of 5 μm thickness was placed on an adherent slide, the paraffin was removed with xylene, and then the tissue sections were soaked in Weigel’s hematoxylin stain for 10 minutes and rinsed in water for 1 minute. Staining was continued with Bibridge Scarlet Acid Violetin for 10 minutes, rinsed with water, incubated with phosphotungstic acid-phosphomolybdic acid for 5 minutes, stained with aniline blue for 10 minutes, and fixed with 1% acetic acid for 2 minutes. Finally, the slides were rinsed with distilled water and dehydrated and covered. After staining, the fibrous tissue appears blue, the cytoplasm red, and the nuclei purple. The percentage of collagen stained area was calculated using Image-Pro Plus 6.0 software. Collagen volume fraction (CVF) was calculated as collagen area/total area.

### Statistical analysis

Western blotting results were analyzed using Image J (version 1.48, National Institutes of Health) software. Matson staining was acquired using an Olympus light microscope. All experimental data were analyzed using SPSS 23.0 (IBM, USA). One-way analysis of variance (ANOVA) and Tukey test were used and histograms were generated using GraphPad Prism 5.01 software. *P < 0.05 and **P < 0.01 were considered significant.

## Results

### Aerobic exercise training mitigated cardiac dysfunction after MI

**Fig 2:**
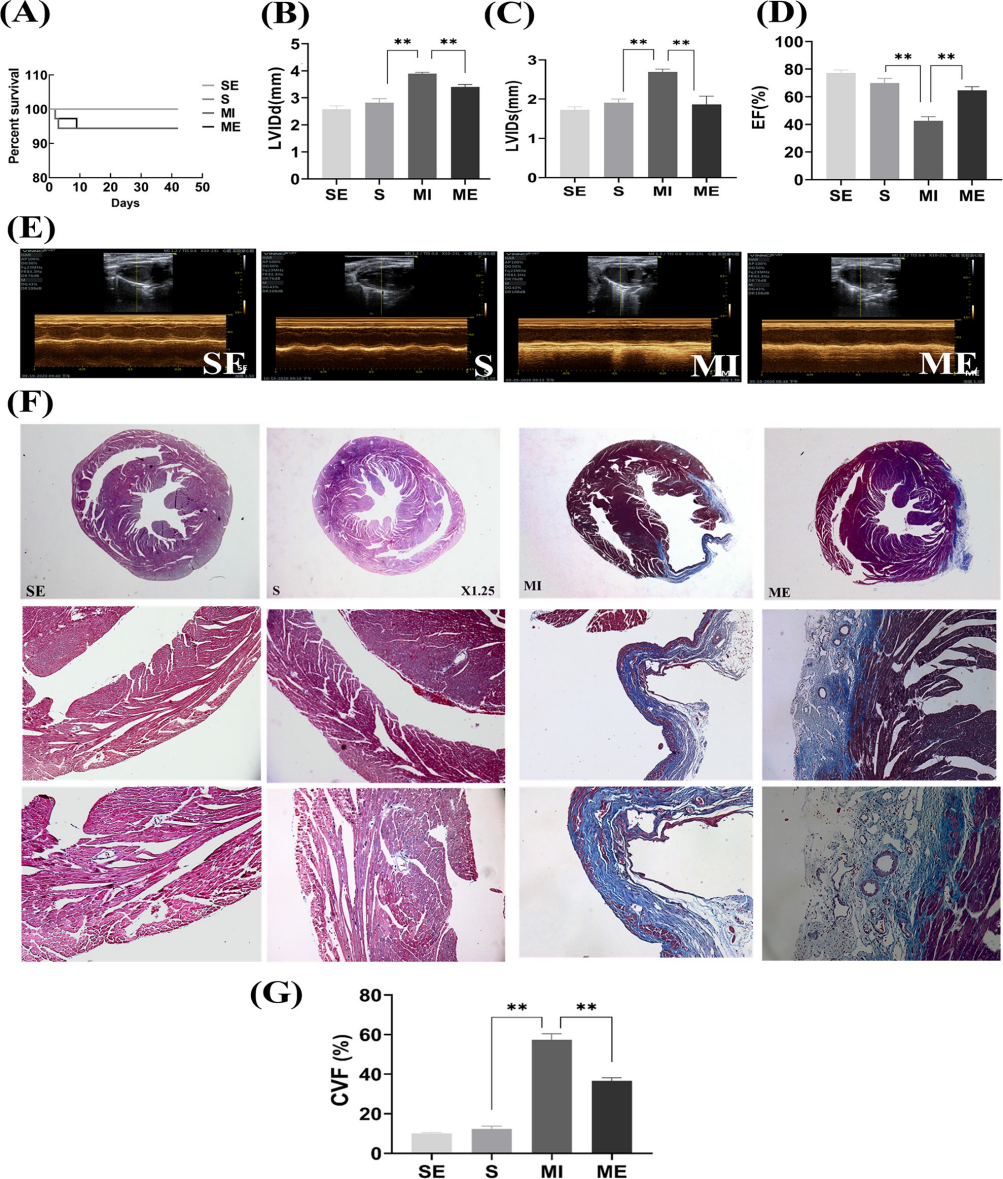
Aerobic exercise improves infarction-induced cardiac dysfunction. (A) Survival curve of mice in different groups, n = 8–10. Systolic and diastolic function of the left ventricle is assessed by measuring small animal ultrasound parameters, including (B) LVIDd, (C) LVIDs, (D) EF and (E) Ultrasound image acquisition (n = 6). Light microscope capture of Masson stained image with collage in the blue area, normal myocardial myofibrils in the red area, and nuclei in the dark brown area (scale bar = 0.61 μm). (F) Masson stained image capture results (G) Collagen volume fraction (CVF %) determined by digital measurement of Matson staining (n = 3). SE, S+ exercise group; S, sham group; MI, myocardial infarct group; ME, MI+ exercise group. *P<0.05, **P<0.01.

It has been well demonstrated that aerobic exercise significantly improves the changes in myocardial function after myocardial infarction. Therefore, we assessed the cardiac function of mice by small animal ultrasound. Compared with the S group, mice in the MI group had significantly decreased EF (*P*<0.01) and significantly increased both LVIDd and LVIDs (*P*<0.01). Compared with the MI group, EF (*P*<0.01) was significantly increased and both LVIDd and LVIDs (*P*<0.01) were significantly decreased in the ME group (Fig 2). The results suggest that aerobic exercise has a significant protective effect on cardiac function after MI.

Next, we further used Masson’s trichrome staining to determine the extent of myocardial fibrosis. We did not observe myocardial infarction in sham-operated mice in the sedentary and exercise groups. The surgery resulted in significant myocardial infarction in mice in the sedentary and exercise groups compared to mice in the corresponding sham-operated groups, as evidenced by large areas of blue-stained myocardial fibrosis. Notably, myocardial fibrosis was significantly reduced in mice that underwent five weeks of aerobic exercise training compared to quiescent mice. Collagen volume fraction (CVF %) in cardiac sections, quantified by Masson staining (Fig 2), was significantly reduced during exercise training in MI mice.

### Aerobic exercise regulated the expression of miR-133a-3p and CTGF following MI

**Fig 3:**
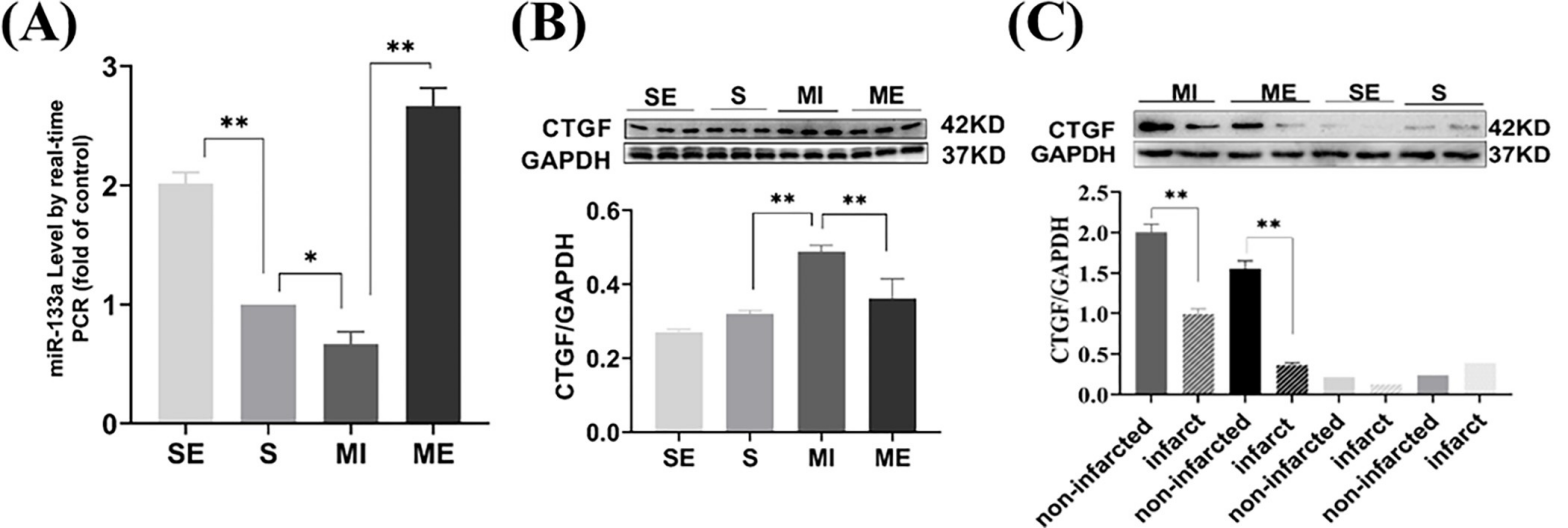
The expression of miR-133a-3p and CTGF in cardiac muscle. (A) miR-133a-3p mRNA expression in the heart (n = 3). (B) Western blotting results of CTGF protein in heart tissues (n = 3). (C) Western blot images of CTGF protein in infarct area and non-infarct area in different groups (n = 3). The values are presented as the mean ± SEM. SE, S+ exercise group; S, sham group; MI, myocardial infarct group; ME, MI+ exercise group. *P<0.05, **P<0.01.

Then, to determine whether aerobic exercise affects miR-133a-3p and CTGF expression in MI mice, we examined the parameters in the hearts of all mice using RT-qPCR and western blotting. As shown in Fig 3, we found that miR-133a-3p levels (*P*<0.05) were significantly decreased and CTGF (*P*<0.01) expression was significantly increased in the MI group compared to the S group, which was significantly reversed by aerobic exercise (*P*<0.01). We also examined the protein expression levels of CTGF in the infarcted and non-infarcted regions of the heart. It was found that CTGF expression was significantly up-regulated in the infarcted region in both the MI and ME groups compared with the non-infarcted region, while there was no significant change between the two regions in the S and SE groups. Taken together, these results suggest that miR-133a-3p and CTGF play a key role in exercise-induced cardioprotection.

### Aerobic exercise inhibited MI-induced cardiomyocyte apoptosis

**Fig 4:**
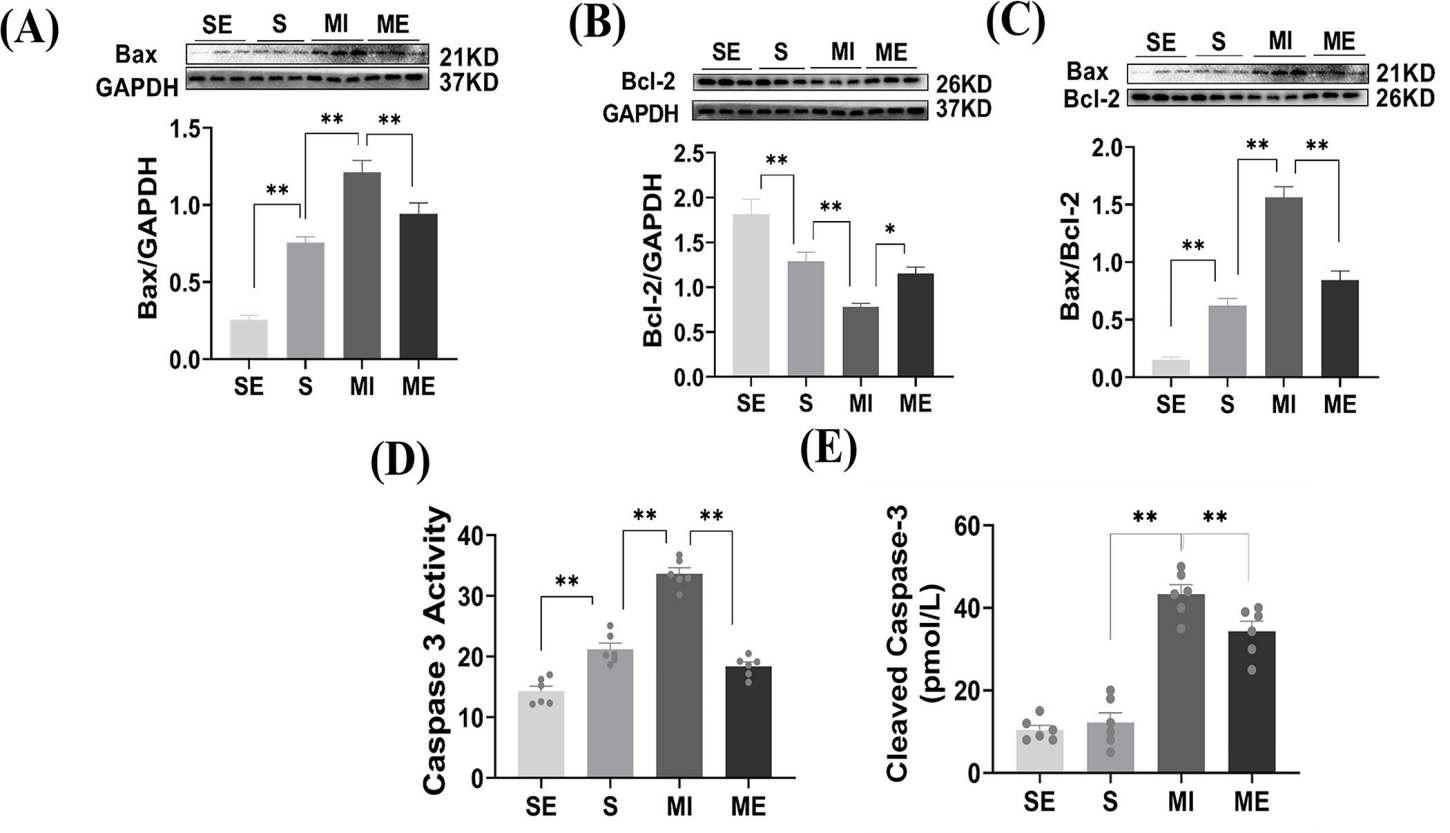
The ameliorative effect of aerobic exercise on myocardial infarction-induced apoptosis in cardiomyocytes. (A, B) Western blotting results of apoptosis-related proteins Bax and Bcl-2 in myocardial tissues (n = 3). (C) BAX/Bcl-2 protein blotting ratio. (D-E) Relative activity of Caspase-3 and Cleaved Caspase-3 enzyme in myocardial tissue using enzyme markers (n = 6). The values are presented as the mean ± SEM. SE, S+ exercise group; S, sham group; MI, myocardial infarct group; ME, MI+ exercise group. *P<0.05, **P<0.01.

We proceeded to investigate whether exercise-induced cardioprotection was associated with a decrease in apoptosis levels using Western blotting and kits. The experimental data showed that apoptosis-related protein (Bax) expression was confirmed to be significantly increased and Bcl-2 expression and Bax / Bcl-2 ratio was significantly decreased in the MI group compared with S group, which was significantly improved by 5 weeks of aerobic platform running exercise, as shown in Fig 4A–4C. Similar to this MI-induced increase in apoptotic protein levels, analysis of Caspase-3 and Cleaved Caspase-3 activity showed that MI also induced a significant increase in Caspase-3 and Cleaved Caspase-3 activity in the hearts of resting mice, as shown in Fig 4D and 4E. However, exercise training attenuated this MI-induced increase in myocardial Caspase-3 and Cleaved Caspase-3 activity. In conclusion, these findings, as well as protein and enzyme changes, suggest that 5 weeks of exercise training alleviates MI-induced myocardial dysfunction by inhibiting MI-induced apoptosis.

### AICAR regulated the expression of miR-133a-3p and CTGF following MI

**Fig 5:**
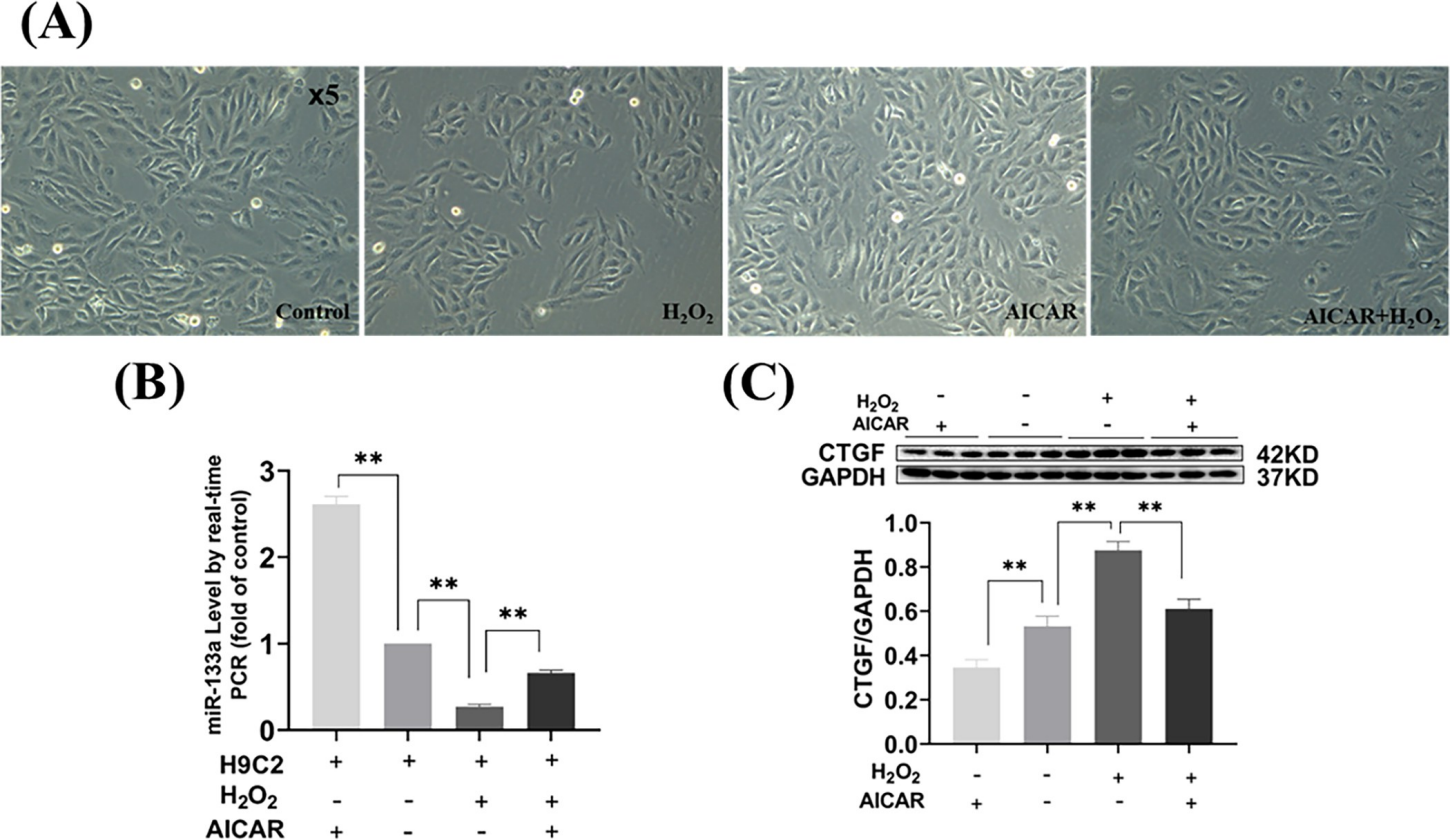
The expression of miR-133a-3p and CTGF in H9C2 cells. (A) H9C2 cells morphology under light microscope. (B) Quantitative reverse transcription polymerase chain reaction (qRT-PCR) analysis of miR-133a-3p expression in the H9C2 cells. (C) Western blot images of CTGF protein in H9C2 cells and densitometric analysis of the band. The values are presented as the mean ± SEM. *P<0.05, **P<0.01.

Light microscopy observed that H9C2 cells in the control and AICAR groups grew against the wall, were myoblast-like, long and dense, with clear contours and strong refractive cell edges (Fig 5A).

Western blotting results showed that miRNA-133a expression was significantly decreased and CTGF expression was significantly up-regulated in the H_2_O_2_ intervention group compared with the control group (*P*<0.01); miRNA-133a expression was significantly up-regulated and CTGF expression was significantly down-regulated in the AICAR intervention group (*P*<0.01) compared with the control group; compared with the H_2_O_2_ intervention group compared with the H_2_O_2_ intervention group; miRNA-133a expression was significantly up-regulated and CTGF expression was significantly down-regulated in H9C2 cells after AICAR intervention (*P*<0.01, Fig 5B and 5C). This indicates that exercise played an important role in upregulating miRNA-133a and downregulating CTGF expression.

### miRNA-133a inhibits H2O2-induced CTGF in H9C2 cardiomyocytes through CTGF

**Fig 6:**
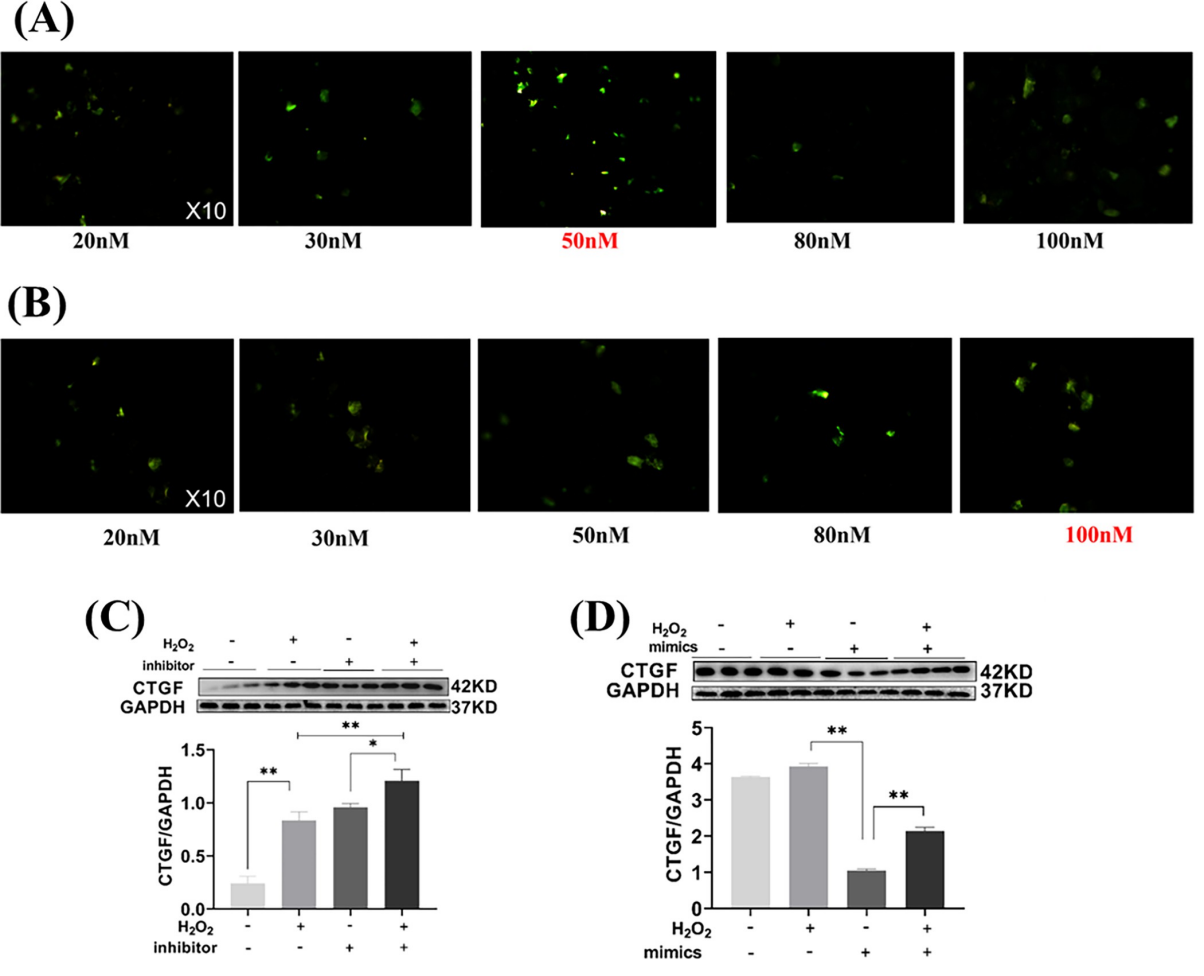
The expression of CTGF in miR-133a-3p inhibitor and mimics transfected H9C2 cells. (A) Transfection concentrations of miR-133a-3p inhibitor in H9C2 cells. (B) Transfection concentrations of miR-133a-3p mimics in H9C2 cells. (C) Expression of CTGF in H9C2 cells after transfection with miR-133a-3p inhibitor. (D) Expression of CTGF in H9C2 cells after transfection with miR-133a-3p mimics. The values are presented as the mean ± SEM. *P<0.05, **P<0.01.

For miR-133a-3p mimics and miR-133a-3p inhibitor, we transfected H9C2 cells at different concentrations of 20 nM, 30 nM, 50 nM, 80 nM and 100 nM, respectively. And immunofluorescence staining revealed that the optimal transfection concentration for miR-133a-3p inhibitor was at a concentration of 50 nM, miR-133a-3p mimics at 100 nM (Fig 6A and 6B).

The results of western blotting experiments showed that CTGF expression was significantly upregulated in the H_2_O_2_ group compared with the contol group (*P*<0. 01), while miR-133a-3p inhibitor could further increase the increase of CTGF expression (*P*<0.05), while CTGF expression was significantly decreased in the miR-133a-3p mimics group compared with the H_2_O_2_ group (*P*<0. 01). It showed that miR-133a-3p significantly decreased the H_2_O_2_-induced CTGF expression.

### AMPK agonist AICAR and miRNA-133a intervention inhibits H2O2-induced apoptosis in H9C2 cardiomyocytes

**Fig 7:**
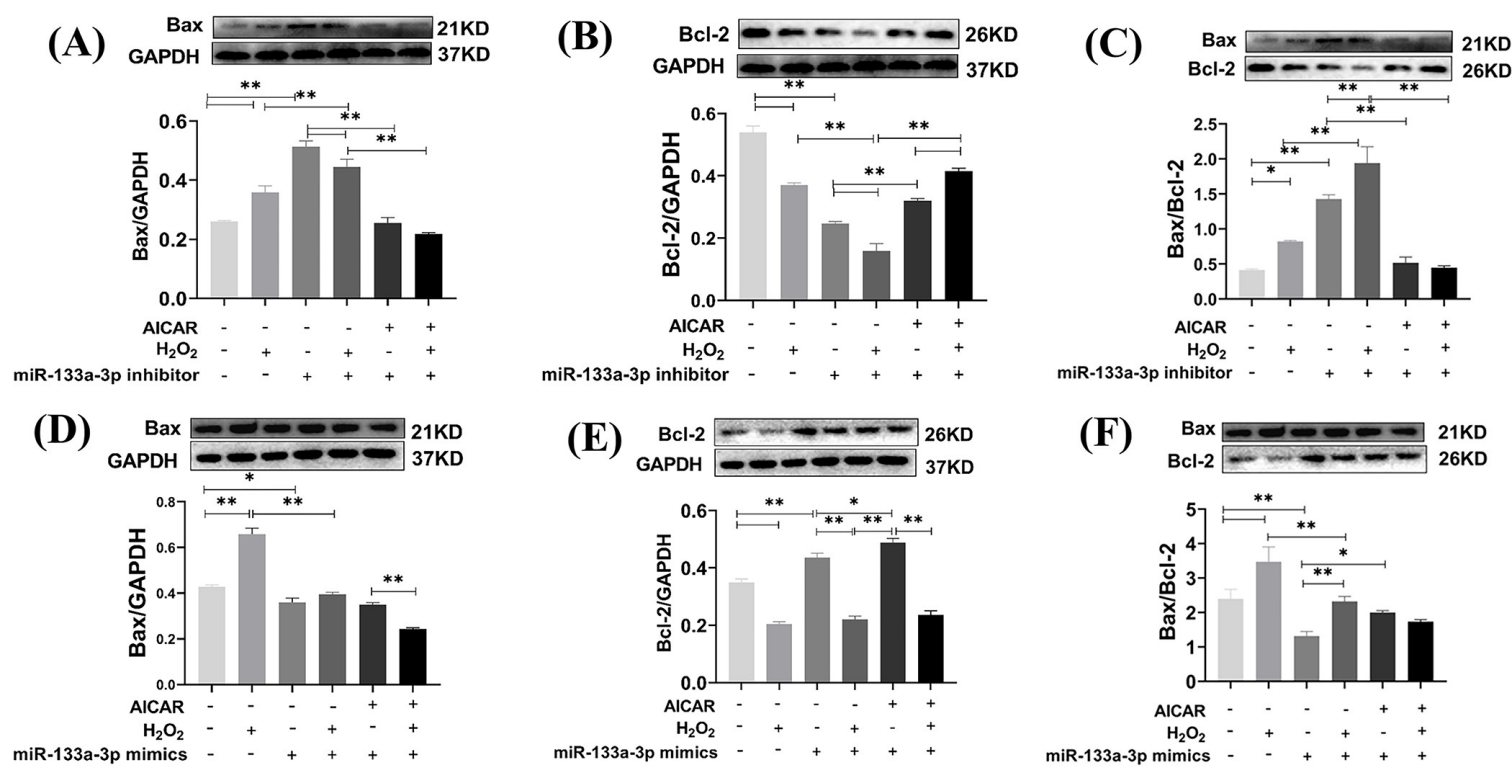
The expression of apoptosis in miR-133a-3p inhibitor and miR-133a-3p mimics transfected H9C2 cells. (A) Expression of apoptosis in H9C2 cells after transfection with miR-133a-3p inhibitor. (B) Expression of apoptosis in H9C2 cells after transfection with miR-133a-3p mimics. The values are presented as the mean ± SEM. *P<0.05, **P<0.01.

Western blotting results showed that Bax expression and Bax/Bcl-2 ratio was significantly up-regulated, and Bcl-2 expression was severely down-regulated, in the miR-133a-3p inhibitor intervention group compared with the control group (*P*<0.01), and AICAR can significantly reverse this phenomenon (*P*<0. 01, Fig 7A–7C).

Bax and Bax/Bcl-2 expression was significantly reduced and Bcl-2 expression was significantly increased in the miR-133a-3p mimics group compared to the normal group (*P*<0.01). Compared with the H_2_O_2_ group, miR-133a-3p mimics significantly reduced H2O2-induced Bax and Bax/Bcl-2 expression (*P*<0.01), while AICAR further reduced their expression (*P*<0.01, Fig 7D–7F). This suggests that miR-133a-3p mimics significantly inhibited H_2_O_2_-induced apoptosis, while AICAR deepened the cardiomyocyte protective effect of miR-133a-3p mimics.

## Discussion

Various remodeling of cardiac structures such as apoptosis, myocardial hypertrophy, ventricular wall thinning, and myocardial interstitial fibrosis occur after myocardial infarction, which is the common pathological basis of many cardiovascular diseases and a major causative factor of heart failure. The protective effect of exercise on the damaged heart as a cost-effective, non-pharmacologic, and low side-effect rehabilitation tool has received widespread attention from scholars. In the present study, aerobic exercise was confirmed by animal and cellular experiments to be effective in improving MI cardiac function, a report that is consistent with the literature [31, 32]. The health of the cardiovascular system is the result of a combination of factors, whose central myocardial fibers are one of the most important links in the evolution of the heart into heart failure. Myocardial fibers can stiffen the ventricular wall and reduce its function, leading to decreased cardiac compliance, cardiac insufficiency and even failure [32]. The results of this study found that compensatory myocardial fibrosis increased significantly after myocardial infarction, and interestingly, myocardial compensatory fibrosis was significantly lower in the aerobic exercise group compared to MI group. This suggests that aerobic exercise can reduce compensatory fibrosis caused by myocardial infarction.

Apoptosis plays a crucial role in the pathology of myocardial infarction. Excessive apoptosis of cardiomyocytes is the main cause of cardiac dysfunction [33]. In the present study, myocardial Bcl-2 expression was reduced, Bax and Caspase-3 expression was significantly increased in MI mice. There is growing evidence that miRNAs are critical in the regulation of cardiovascular disease, but further studies are needed to investigate the specific molecular mechanisms by which miRNAs regulate apoptosis and to identify their direct and indirect targets. Studies have demonstrated the critical role of miRNAs in MI affecting apoptosis, and downregulation of miR-17-5p improves cardiac function in MI by inhibiting apoptosis of endothelial cells [34]. In a rat infarction model, upregulation of miR-298 significantly reduced the expression of BAX, cytochrome-c and cleaved caspase-3, reduced myocardial apoptosis and improved infarcted cardiac function [35]. miR-133a is widely present in the normal heart, is significantly altered when heart is injured, and plays a very important role in recovery from acute myocardial infarction. However, the delivery of miR-133a to the site of action remains a challenge. It has been reported that miR-133 protects the heart by actually ameliorating cardiac injury, reducing myocardial infarct size, and inhibiting cardiomyocyte apoptosis, inflammation, and oxidative stress through the SIRT3/AMPK pathway [36]. Consistent with the results of our present study, miR-133a-5p was found to be significantly decreased after MI and 5 weeks of aerobic exercise can significantly upregulate its expression on mice. We used in vitro cell culture experiments and found that after transfection of H9C2 rat cardiomyocytes with miR-133a-5p mimics and inhibitors, the expression of CTGF and Bax was significantly reduced, and Bcl-2 expression was significantly increased, whereas apoptosis was significantly increased after transfection with miR-133a-5p mimics and intervention with miR-133a-5p inhibitors. Zhang et al. found that miR-133 was under-expressed in a mouse model of myocardial infarction, whereas overexpression of miR-133 significantly improved myocardial infarction mice s cardiac function index and exercise function and reduced the area of myocardial infarction [37]. Habibi et al. found that exercise training significantly decreased the expression of Bax, Caspase-3, and caspase-8, increased the expression of miR-133 and Bcl-2 and anti-apoptotic markers, and decreased apoptotic biomarkers in warmed ovariectomized rats [38]. Studies have shown that microRNAs can regulate myocardial remodeling from multiple transduction pathways including connective tissue growth factor (CTGF), angiotensin system and inflammatory factors [17, 18]. CTGF is a compliant growth factor that serves as a diagnostic marker and therapeutic target for cardiac fibrosis and heart failure. miR-133 is a strong negative regulator of CTGF expression in cardiac factors [23].

## Conclusions

In conclusion, the present study showed that aerobic exercise inhibited infarct-induced cardiomyocyte apoptosis by upregulating miR-133a-5p and downregulating CTGF expression. This study provides what we believe are new insights into the mechanisms of exercise-induced cardiac repair. miR-133a-5p and CTGF could be considered as potential markers of MI disease. In the present study, we did not generate CTGF knockout animals and did not evaluate the CTGF signaling pathway, which would provide more direct and reliable evidence. Therefore, our team will use CTGF knockout animals to further validate the upstream and downstream relationships of the key cardioprotective mediators identified in this study. In addition, CTGF has multiple functions in cellular activity, including apoptosis, cellular inflammation, and angiogenesis. Exercise is known to improve cardiac function by reducing inflammation and promoting neovascularization. We will further investigate whether exercise can improve the function of infarcted hearts by reducing inflammation and promoting neovascularization through the CTGF pathway. All these findings may eventually reveal effective non-pharmacological and pharmacological exercise strategies to manage and promote recovery of MI patients.

## Supporting information

S1 Raw images (PDF)

S1 Dataset (XLSX)
