# Supplementary material for: Aerobic exercise protects MI heart through miR-133a-3p downregulation of connective tissue growth factor
Source: PLoS One. 2024 Jan 25;19(1):e0296430. doi: 10.1371/journal.pone.0296430 (PMC10810442; doi:10.1371/journal.pone.0296430)
Supplement: S1 Raw images — (PDF) [file pone.0296430.s001.pdf]

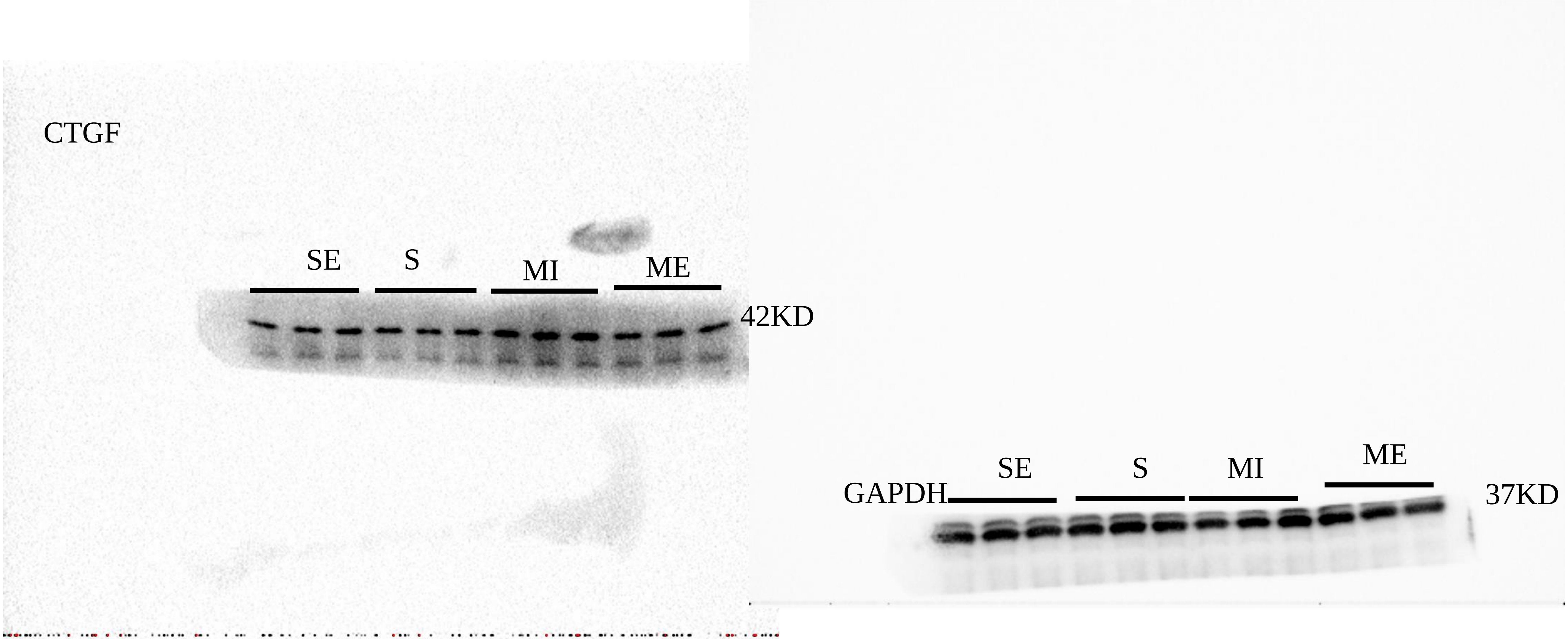

Figure 3. The expression of miR-133a-3p and CTGF in cardiac muscle.

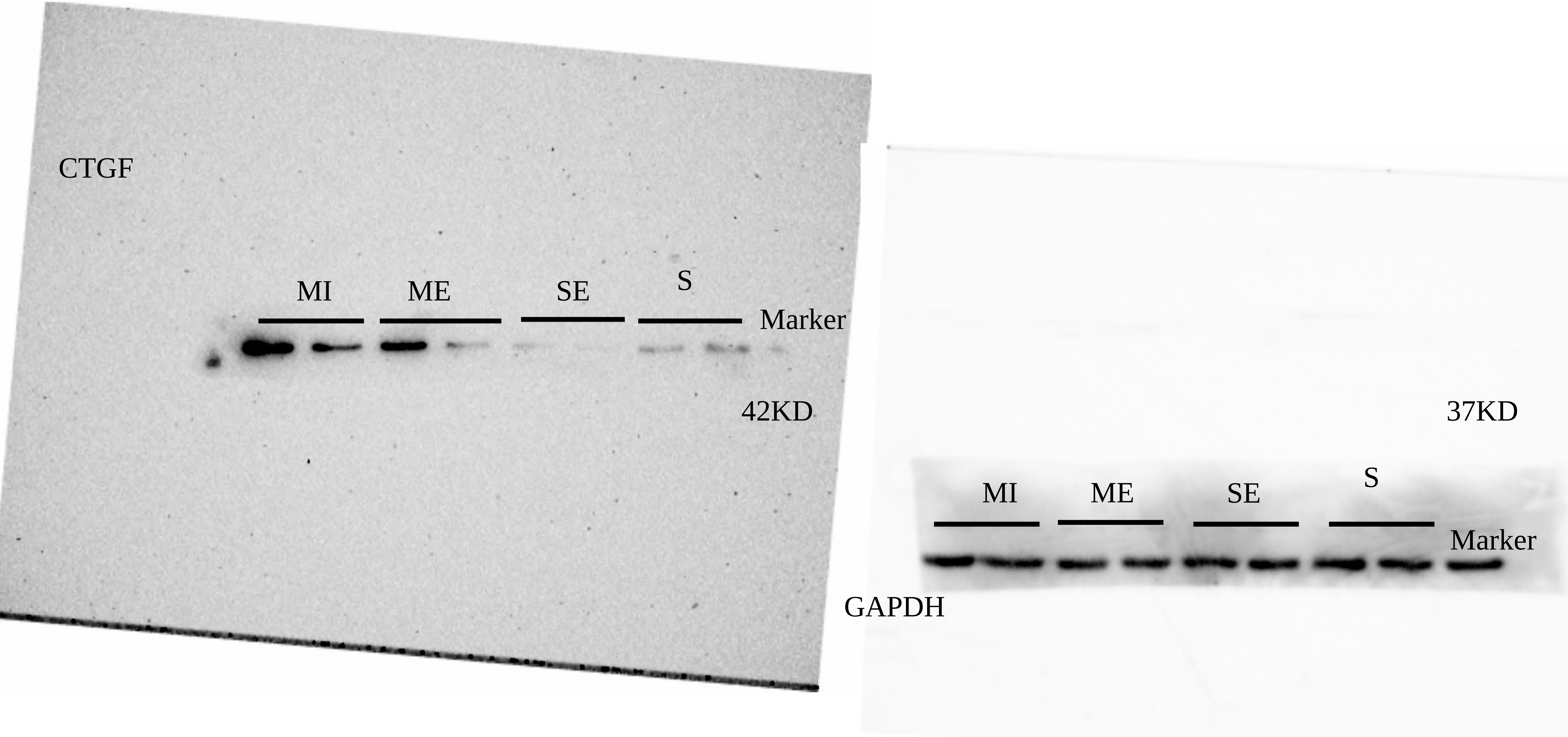

Figure 3. The expression of miR-133a-3p and CTGF in cardiac muscle.

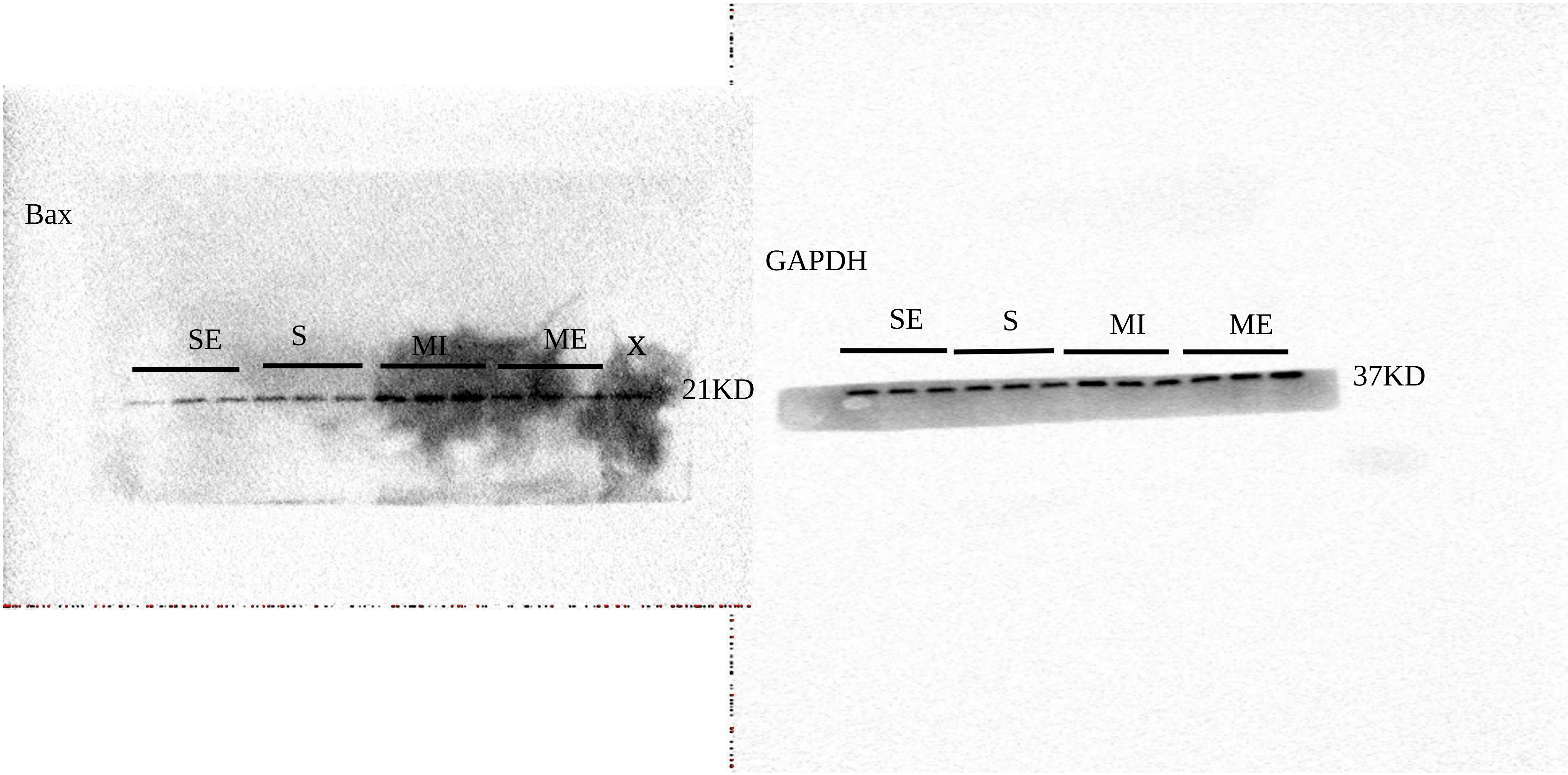

Figure 4. Effect of exercise on cardiomyocyte apoptosis following MI.

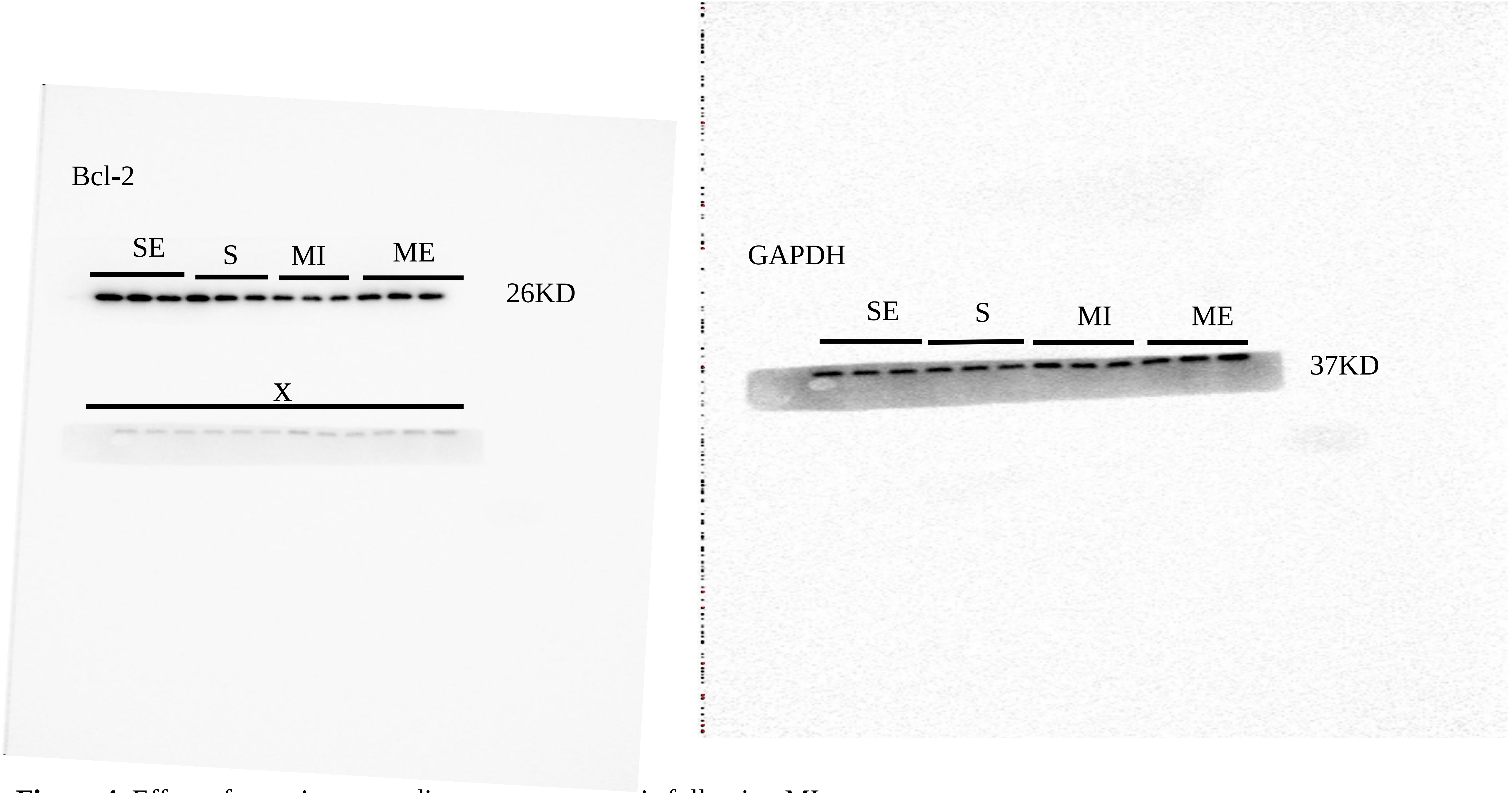

Figure 4. Effect of exercise on cardiomyocyte apoptosis following MI.

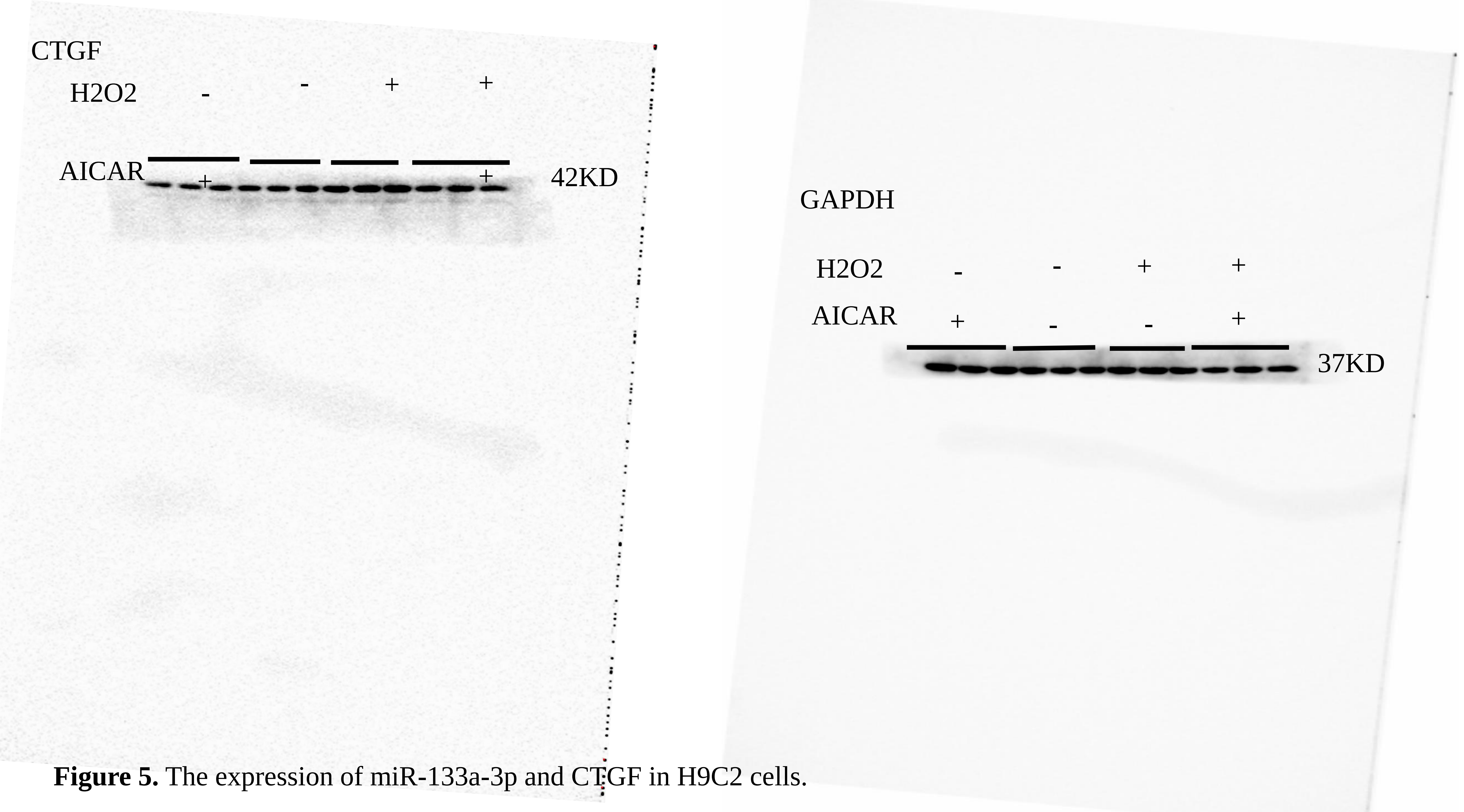

Figure 5. The expression of miR-133a-3p and CTGF in H9C2 cells.

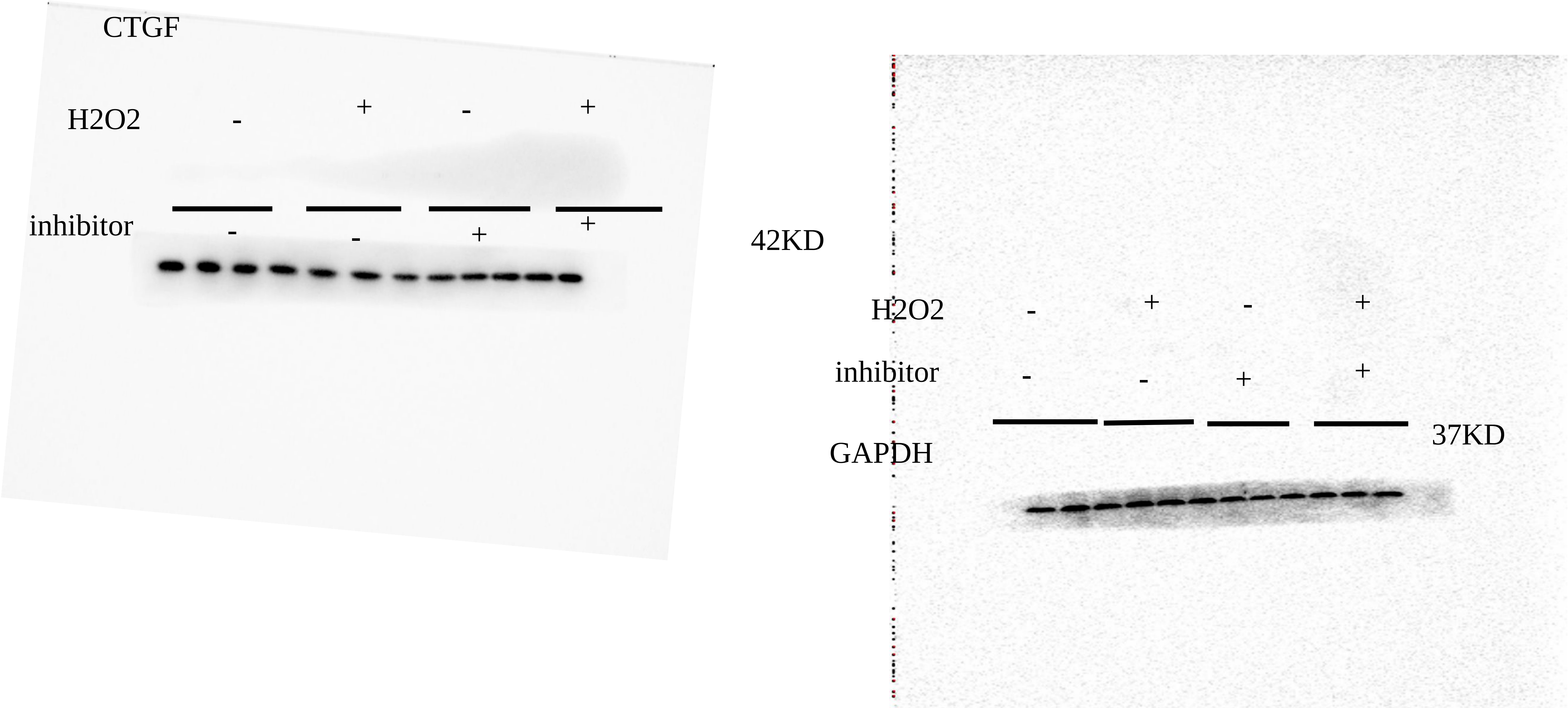

Figure 6. The expression of CTGF in miR-133a-3p inhibitor and mimics transfected H9C2 cells.

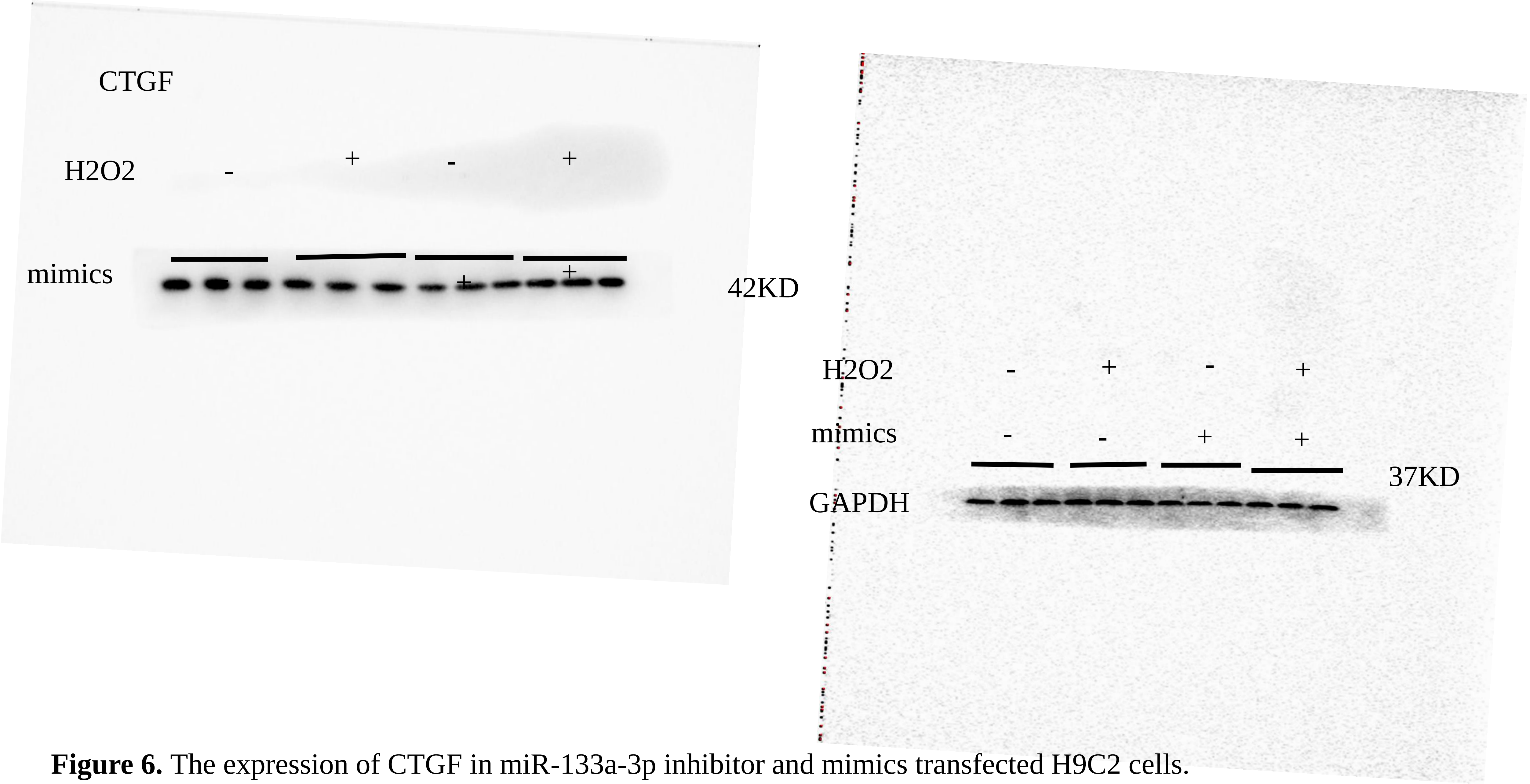

Figure 6. The expression of CTGF in miR-133a-3p inhibitor and mimics transfected H9C2 cells.

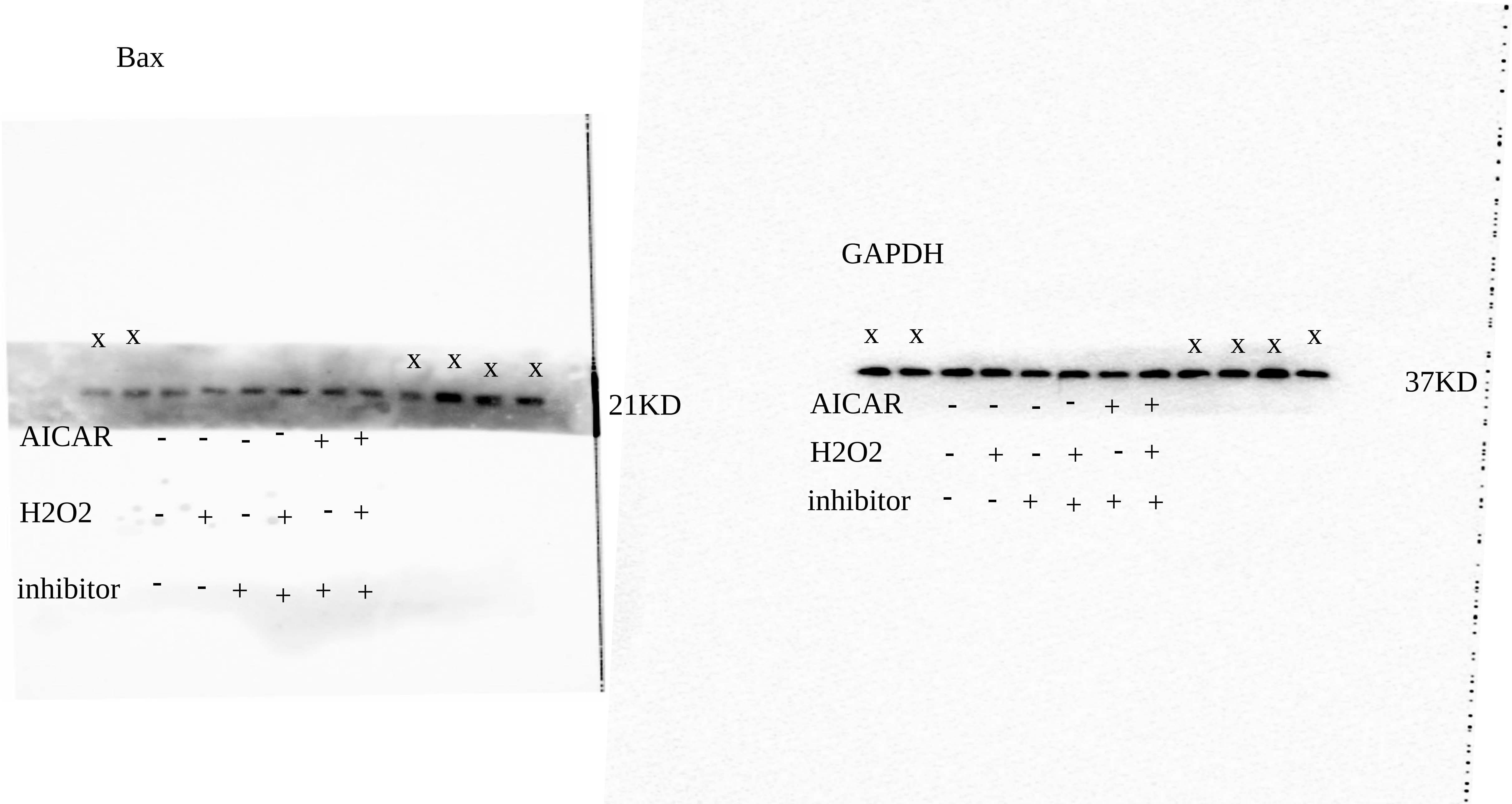

Figure 7. The expression of apoptosis in miR-133a-3p inhibitor and mimics transfected H9C2 cells.

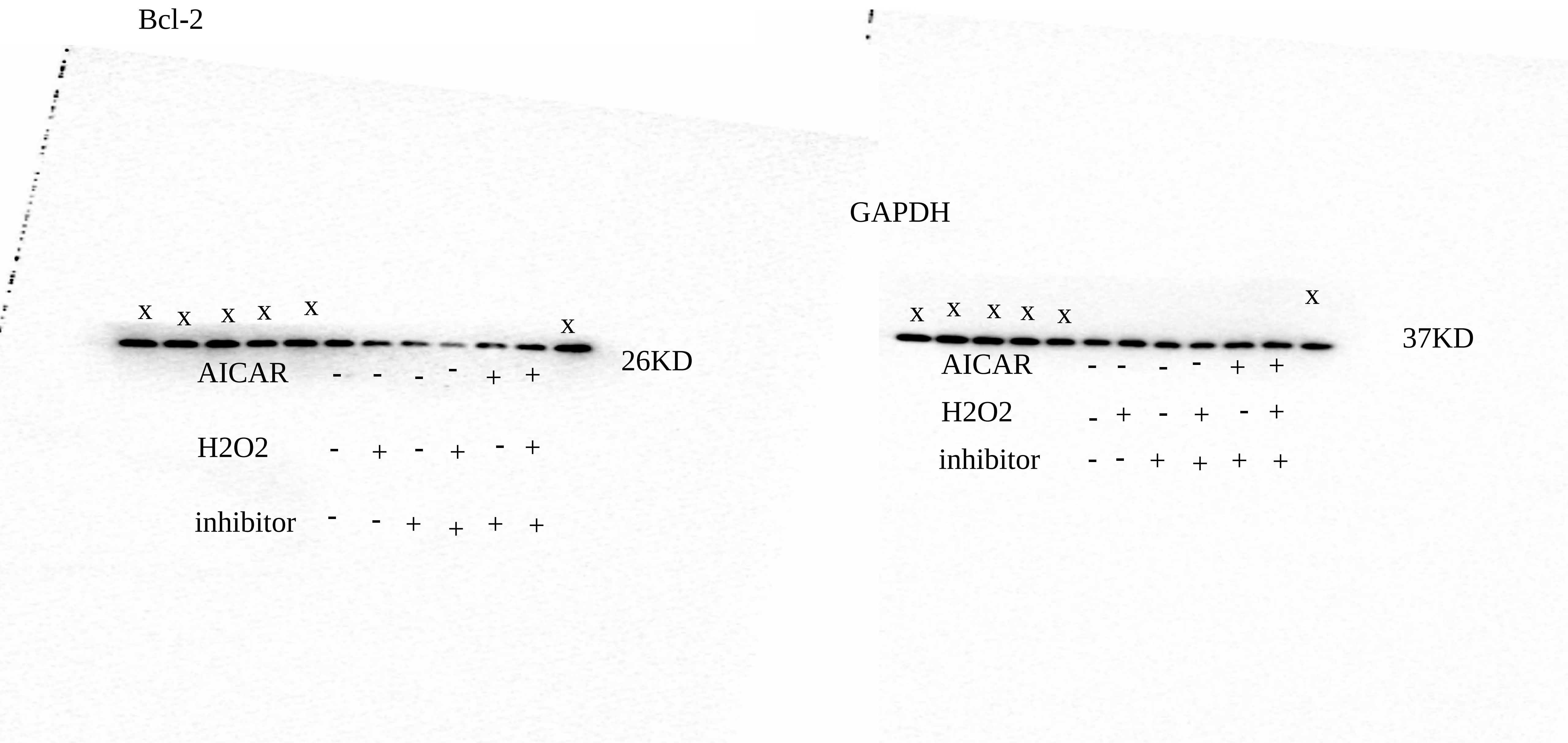

Figure 7. The expression of apoptosis in miR-133a-3p inhibitor and mimics transfected H9C2 cells.

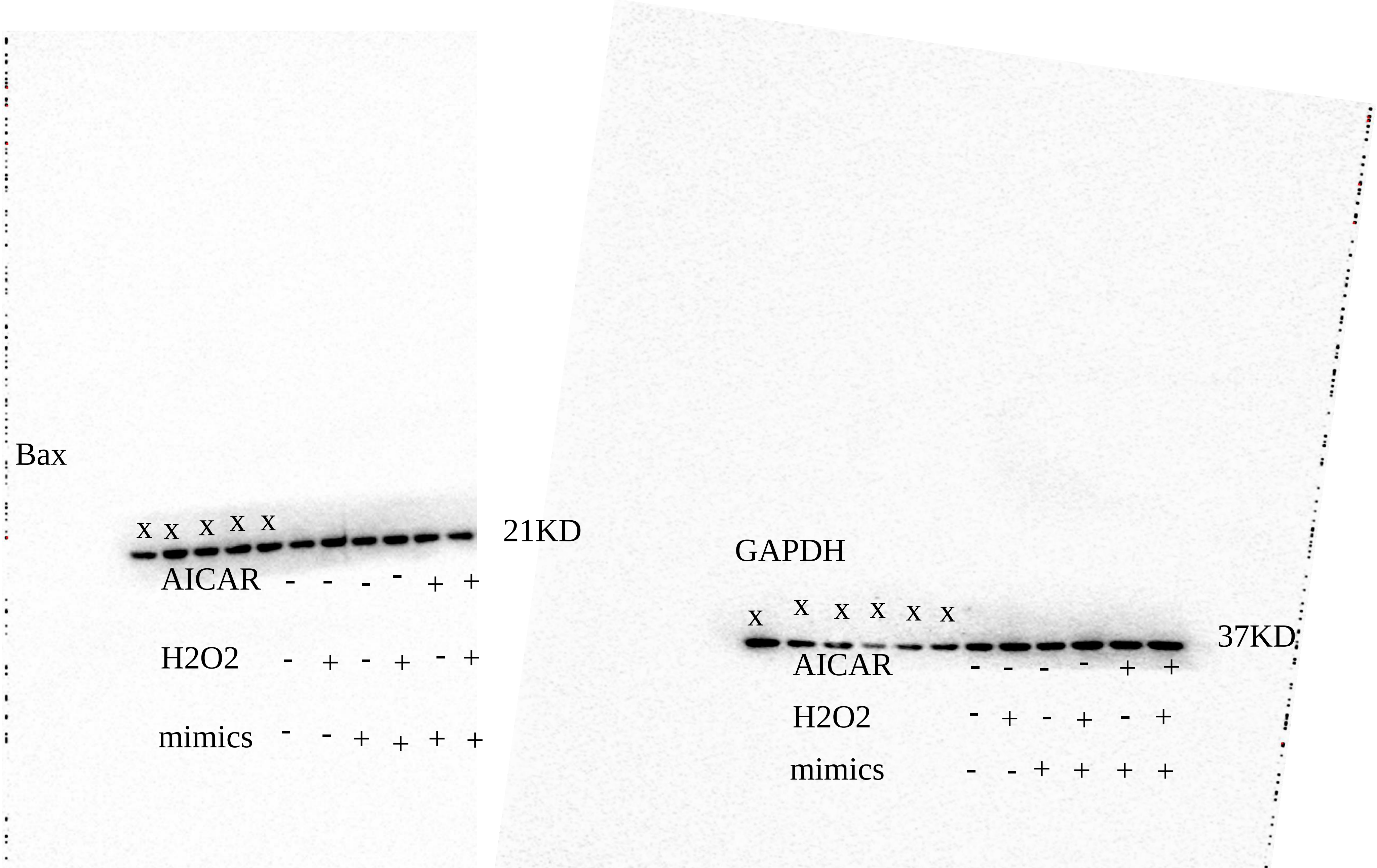

Figure 7. The expression of apoptosis in miR-133a-3p inhibitor and mimics transfected H9C2 cells.

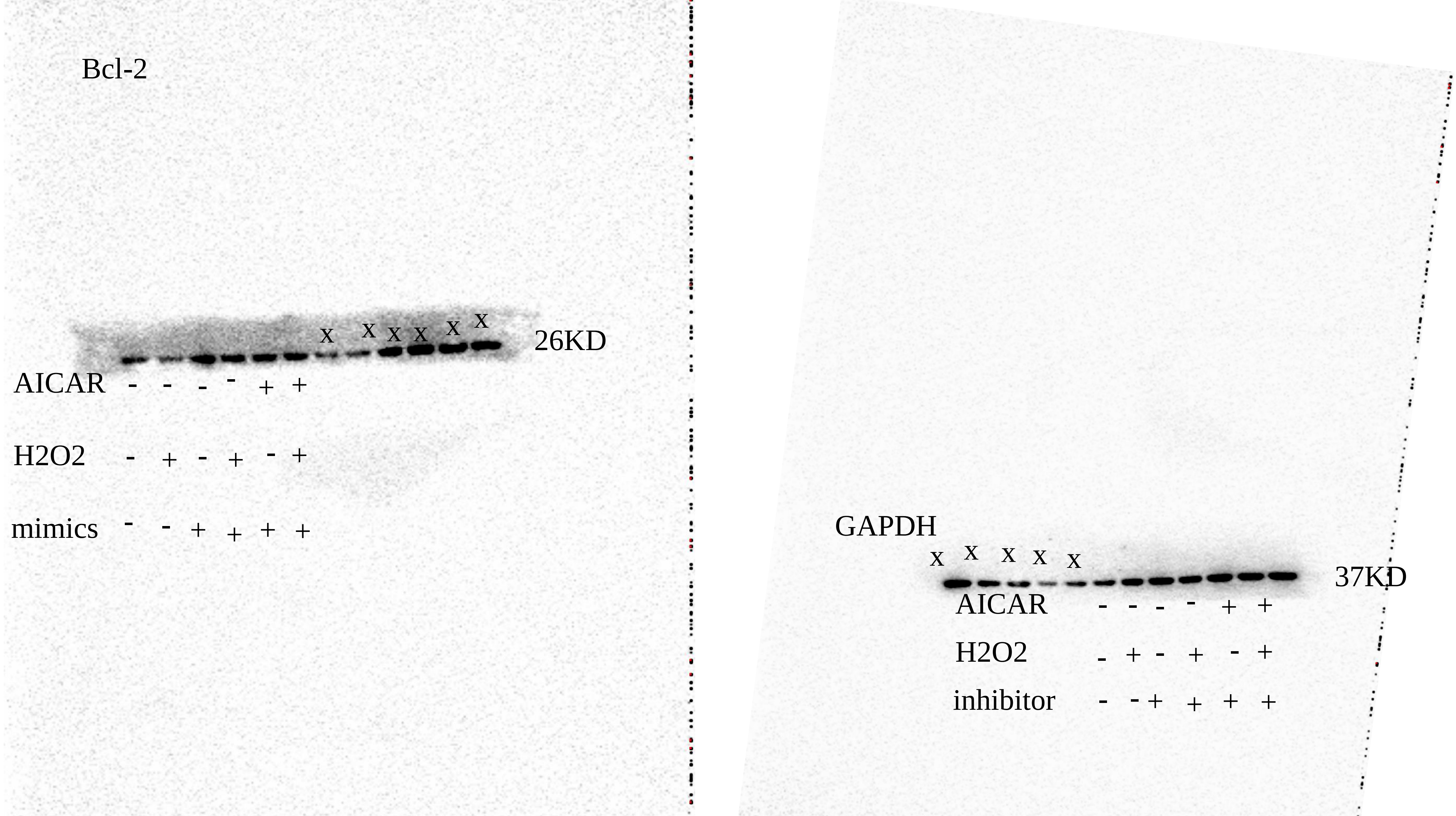

Figure 7. The expression of apoptosis in miR-133a-3p inhibitor and mimics transfected H9C2 cells.
